# Severe macular complications in glaucoma: high-resolution multimodal imaging characteristics and review of the literature

**DOI:** 10.1186/s12886-023-03068-z

**Published:** 2023-07-14

**Authors:** Hugo Lama, Michel Pâques, Emmanuelle Brasnu, Jade Vu, Céline Chaumette, Bénédicte Dupas, Christine Fardeau, Ismaël Chehaibou, Jean-François Rouland, Guillaume Besombes, Marc Labetoulle, Antoine Labbé, Antoine Rousseau

**Affiliations:** 1 Department of Ophthalmology, Assistance Publique - Hôpitaux de Paris - Université Paris Saclay, 78, rue du Général Leclerc, Le Kremlin Bicêtre, 94275 France; 2 Department of Ophthalmology, Quinze-Vingts National Eye Hospital, IHU Foresight, Paris, France; 3 Ophtalmopôle Cochin, APHP, Paris, France; 4 Department of Ophthalmology, Pitié Salpétrière Hospital, APHP, Paris, France; 5 Department of Ophthalmology, Hôpital Claude Huriez, CHRU, Lille, France

**Keywords:** Adaptive optics, Glaucoma, High-resolution imaging, Macular edema, Retinoschisis, Serous retinal detachment

## Abstract

**Purpose:**

To describe imaging characteristics of severe macular complications occurring in glaucoma and discuss available treatments.

**Methods:**

Retrospective case series of glaucomatous patients with macular retinoschisis (MR) and/or serous retinal detachment (SRD). Patients underwent a complete ophthalmological examination and multimodal imaging including retinography, SD-OCT, fluorescein and indocyanine green angiography (FA & ICGA) and adaptive optics (AO).

**Results:**

Ten eyes (8 patients) were included. Initial BCVA was 1.04 ± 1.12 logMAR and IOP was 24.0 ± 9.3mmHg. All eyes presented with MR while SRD was present in 5 eyes (5 patients), with a central macular thickness of 573 ± 152 μm. FA and ICGA allowed to exclude leakage in all cases. A focal lamina cribrosa defect (LCD) was found in four eyes (4 patients) using OCT, with AO providing en-face visualization of the defect in one eye. Outer retinal hole was present in 3 eyes (3 patients). No visual improvement or resolution of the macular retinoschisis was observed in eyes with medical or surgical IOP control (N = 9). Vitrectomy with internal membrane limiting peeling and gas tamponade was performed in one eye with good visual results.

**Conclusions:**

Multimodal high-resolution imaging is essential to diagnose severe macular complications associated with advanced glaucoma.

**Supplementary Information:**

The online version contains supplementary material available at 10.1186/s12886-023-03068-z.

## Introduction

In rare cases, patients with glaucomatous optic neuropathy (GON) may present with macular complications such as retinoschisis and/or serous retinal detachment (SRD) [1]. The former is defined as an abnormal splitting of the neurosensory layers of the retina, which can be observed in various etiologies such as high myopia [2], pachychoroid spectrum disease [3], juvenile X-linked retinoschisis [4] and congenital optic disc pit [5]. In glaucoma patients, retinoschisis is most often located in the peripapillary area and is usually asymptomatic [6, 7]. In this context, macular retinoschisis is much rarer. It mostly occurs in severe or advanced GON, can be associated with SRD, and is associated with a poor visual prognosis [1, 8, 9].

One pathophysiological hypothesis to explain these uncommon retinal complications arising from GON involves the existence of an acquired subclinical focal defect within the lamina cribrosa, allowing the passage of fluid toward the subretinal and intra-retinal spaces [1, 3, 10]. Optical coherence tomography (OCT) allows in some cases to identify these defects, which are often associated with an acquired pit of the optic nerve (APON) [9, 10]. However, the visualization of such defects may be challenging [7]. While adaptative optics (AO) can detect microscopic morphological changes in the lamina cribrosa of glaucoma patients [11], the use of this imaging technique has not been reported in this setting.

In this paper, we report a series of patients with GON presenting with macular complications that were studied with high-resolution multimodal imaging, including spectral domain OCT (SD-OCT) and AO. We provide detailed clinical and imaging characteristics, discuss potential pathophysiological mechanisms, and propose a practical therapeutic algorithm based on a comprehensive review of the available literature.

## Patients and methods

This retrospective multicenter study included patients with glaucoma who presented with macular retinoschisis and/or SRD, managed between June 2016 and April 2020. All participants provided their informed consent. The study adhered to the tenets of the declaration of Helsinki and was approved by the Ethics Committee of the French Society of Ophthalmology (IRB00008855 Société Française d’Ophtalmologie IRB#1).

Inclusion and exclusion criteria.

We included adult patients with GON, i.e. with typical glaucomatous excavation of the optic disc with retinal nerve fiber layer (RNFL) defects on OCT, associated with macular retinoschisis and/or SRD on SD-OCT scans, and with peripheral arcuate visual defects corresponding to areas of neuroretinal rim loss. Other potential causes of retinoschisis / SRD were ruled out on clinical and imaging criteria detailed in Table 1. Both primary open angle glaucoma (POAG), juvenile and normal tension glaucoma (NTG) patients were eligible, as long as typical morphological and compatible functional features of GON were present.

**Table 1:** Exclusion criteria for differential diagnosis of retinoschisis / serous retinal detachment. IOP = intraocular pressure, D = diopter, FA = fluorescein angiography, ICGA = indocyanine green angiography, SRD = serous retinal detachment

Differential diagnosis	Clinical criteria	Imaging criteria
Juvenile X-linked retinoschisis	- Family history of retinoschisis - Retinal detachment and/or vitreous hemorrhage - Peripheral retinoschisis - Normal IOP	- Foveoschisis without communication with the optic disc cavity
Congenital optic disc pit	- Typical optic disc pit (round or oval depression inside the optic disc that differs in color from the surronding disc) - History of congenital ocular disease - Normal IOP	- Hypofluorescent lesions in the optic disc in the early phase of FA, with late hyperfluorescence
Myopic foveoschisis	- Spherical equivalent ≤ -6 D and/or axial length ≥ 26 mm	- Absence of myopic staphyloma using OCT.
Pachychoroid spectrum disease	- Steroid intake - Type A personality	- Subfoveal choroidal thickness ≥ 300 μm - Leakage on FA / ICGA - Retinal pigment epitheliopathy / gravitational tracts
Exudative SRD	- Retinal neovascularization - Ocular inflammatory disease	- Leakage on FA / ICGA

### Ophthalmological examination

Best-corrected visual acuity (BCVA) was measured using the Monoyer chart and then converted to logarithm of minimal angle of resolution (logMAR) units for statistical analysis. All patients underwent complete ophthalmological examination and multimodal retinal imaging, including fundus photography, macular and optic nerve SD-OCT (Spectralis, Heidelberg Engineering, Heidelberg, Germany), fluorescein and indocyanine green angiography (Spectralis, Heidelberg Engineering, Heidelberg, Germany), with early, mild and late phase angiograms and AO (protocol detailed below). SD-OCT included serial horizontal macular scans, vertical and horizontal optic disc scans performed using enhanced depth imaging (EDI) mode, and circumpapillary scans. Glaucoma medications and surgical procedures were recorded for each patient.

### Adaptative optics

AO was used for optic disc imaging, with scans focused on the lamina cribrosa, and macular imaging, using the rtx1 camera (Imagine Eyes, Orsay, France). AO uses an 850-nm flashed-flood source to illuminate the region of interest and acquires images equal to 4° × 4° (1.2 mm × 1.2 mm on the retina and optic disc), with a maximum lateral resolution of 2 μm. Real-time video enables fine focusing, after which a series (Z-stack) of images is acquired over 4 s. Examinations were conducted in a dark room to facilitate imaging without pharmacological pupil dilation, with an external fixation target guiding the fellow eye.

### Statistical analysis

The data were collected to ensure patient anonymity. Descriptive analysis was performed with Microsoft Excel (Mac Version 14.4.1; Microsoft Corp., Redmond, WA, USA) and statistical analysis was performed with R version 3.2.0 software. Continuous variables were presented as mean ± standard deviation, and Kruskall-Wallis test was used for quantitative outcomes. Statistical significance was indicated by *p* < 0.05 (2-tailed).

## Results

### Patients

Ten eyes of 8 patients, four men and four women, aged 55.1 ± 17.1 years, were included (Table 2).

**Table 2:** Clinical characteristics of eyes with severe macular complications associated with glaucomatous optic neuropathy. M = male, F = female, OD = right eye, OS = left eye, BCVA = best corrected visual acuity, mon = months, IOP = intraocular pressure, C/D = cup/disc ratio, POAG = primary open angle glaucoma, NTG = normal tension glaucoma, JG = juvenile glaucoma, MD = mean deviation, dB = decibel, LP = light perception, NA = not available, PPV = pars plana vitrectomy, ILMP = internal limiting membrane peeling, GT = gas tamponade, TRAB = trabeculectomy. Asterisks indicate patients treated with glaucoma eye drops

Case #	Age range/ Gender	Eye	Glaucoma type	Initial BCVA (Snellen)	Initial IOP (mmHg)	C/D	Initial MD (dB)	Surgical treatment	Follow-up (mon)	Final BCVA (Snellen)	Final IOP (mmHg)
1	50s/M	OS	POAG	20/400	17*	1	-26.08	-	6	20/400	15*
2	60s/F	OD	POAG	20/4000	40	1	NA	-	10	No LP	17*
3	70s/F	OD	POAG	20/40	25	0.8	NA	-	12	20/40	18*
		OS	POAG	20/40	22	0.7	NA	-	12	20/40	18*
4	50s/M	OD	POAG	20/1000	40	0.9	NA	-	32	20/1000	14*
5	80s/F	OS	NTG	20/1000	18*	0.8	NA	PPV/ILMP/GT	6	20/32	15*
6	40s/F	OS	NTG	20/25	13	0.9	-16.04	-	8	20/25	12*
7	30s/M	OD	JG	20/25	18*	1	-23.62	-	24	20/32	13*
		OS	JG	20/20	27*	1	-22.76	TRAB	24	20/20	9
8	40s/M	OD	JG	20/80	20*	0.9	-27.22	-	5	20/80	18*

**Fig. 1:**
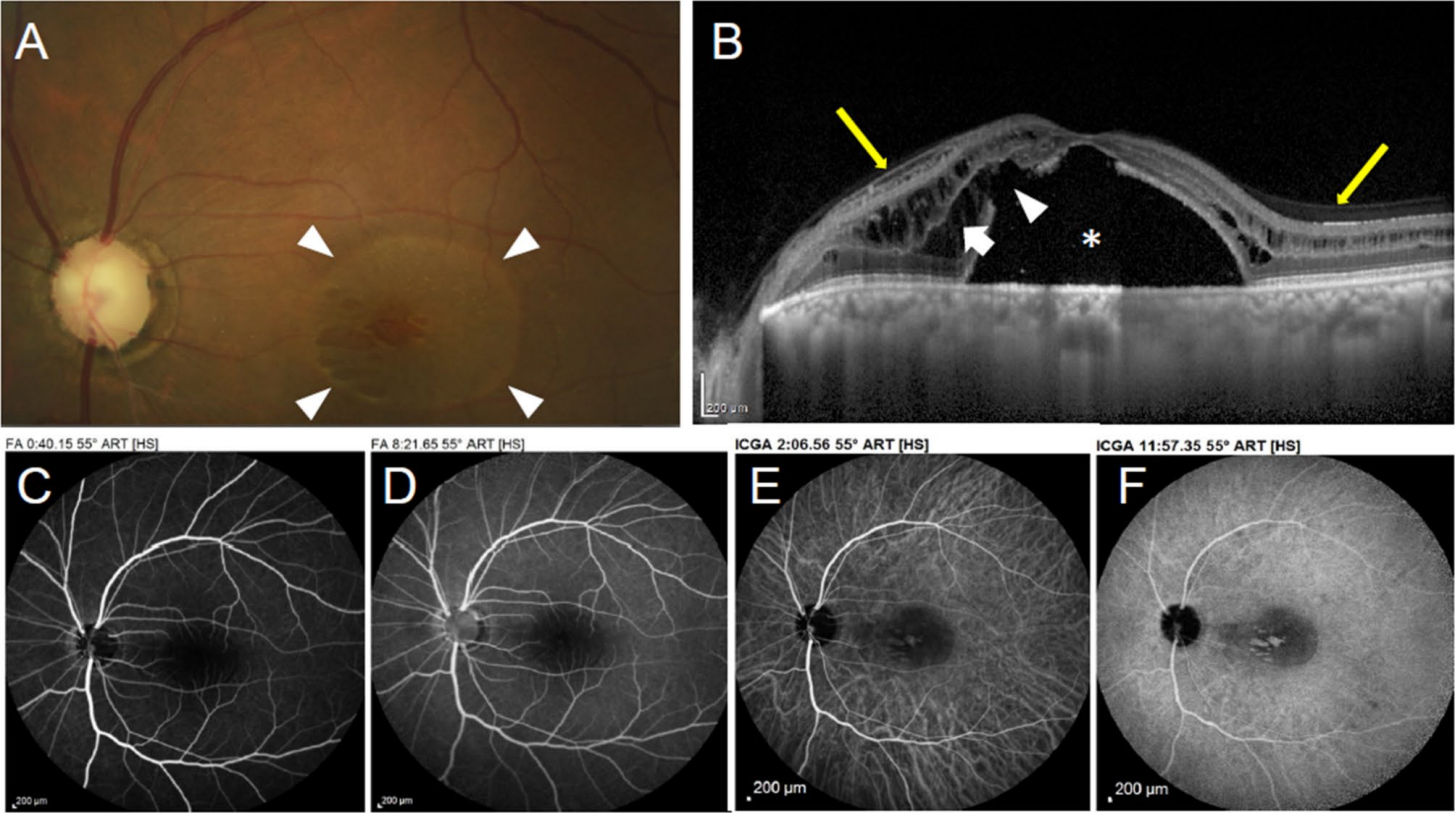
Case #1. **(A)** Color fundus photography of the left eye revealing retinoschisis associated with SRD (arrowheads) and complete optic disc cupping. **(B)** SD-OCT scans across the macula showing macular retinoschisis (arrow) with massive SRD (*) and an outer retinal hole (arrowhead). Yellow arrows highlight the absence of VM traction, with posterior hyaloid membrane remaining parallel to the retina, without conical pattern. **C, D)** Early and late phase fluorescein angiograms & **E, F)** early- and late-phase indocyanine green angiogram showing no leakage

At inclusion, the mean intraocular pressure (IOP) was 24.0 ± 9.3 mmHg, and mean BCVA was 1.0 ± 1.1 logMAR (2.7–0 logMAR). POAG accounted for 5 eyes (4 patients), NTG for 2 eyes (2 patients) and juvenile glaucoma for 3 eyes (2 patients). The mean cup-to-disc ratio (C/D) was 0.9 ± 0.1, and central macular thickness (CMT) was 573 ± 152 μm. In patients for whom Humphrey VF was performed in 5 eyes (4patients: #1, #6, #7, #8), the mean deviation was − 23.1 ± 4.3 dB. Visual field could not be performed because of poor visual acuity in one case (#2). Goldmann VF were performed in the remaining cases (#2, #4, #5) and showed severe glaucomatous scotomas. Interestingly, one patient received three unsuccessful intravitreal injections of anti-VEGF before he was referred for second opinion (case # 1, Fig. 1A-F).

### Multimodal imaging

**Fig. 2:**
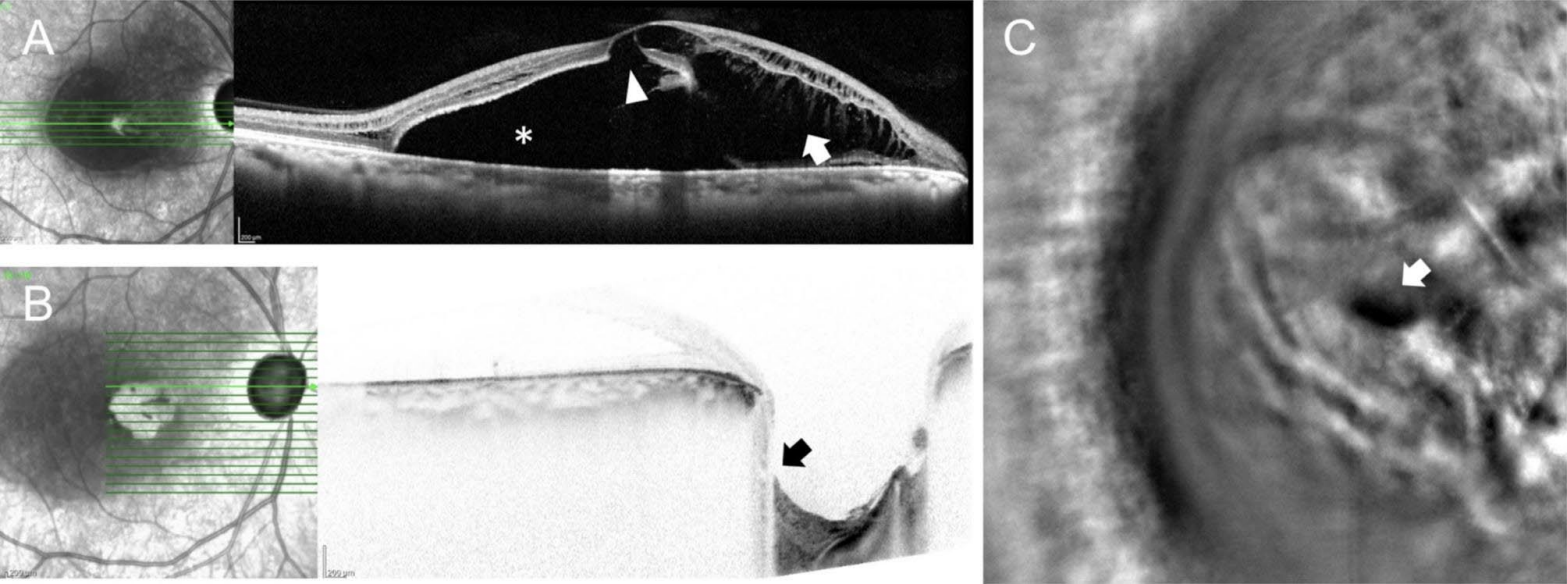
Case #4. **(A)** SD-OCT scan showing macular retinoschisis (arrow) associated with SRD (*) and an outer retinal hole (arrowhead). **(B)** Lamina cribrosa defect on enhanced depth imaging SD-OCT scan (arrow), and **(C)** using AO imaging (white arrow)

OCT confirmed macular retinoschisis in all cases, combined with SRD in 5 eyes (5 patients). An outer retinal hole was present in 3 eyes (3 patients, Figs. 1B and 2 A). FA and ICGA showed no leakage and allowed to rule out choroidal neovascularization, or central serous chorioretinopathy. In all patients, SRD did not fill with dye. However, late ICG filling of cystic-like spaces of the split retina occurred in one patient (Fig. 1F). A LCD was found in 4 eyes (4 patients) using EDI-OCT of the optic nerve, which appeared as a discontinuity in the lamina cribrosa, located temporally or superotemporally in all cases (Fig. 2B, C).

**Fig. 3:**
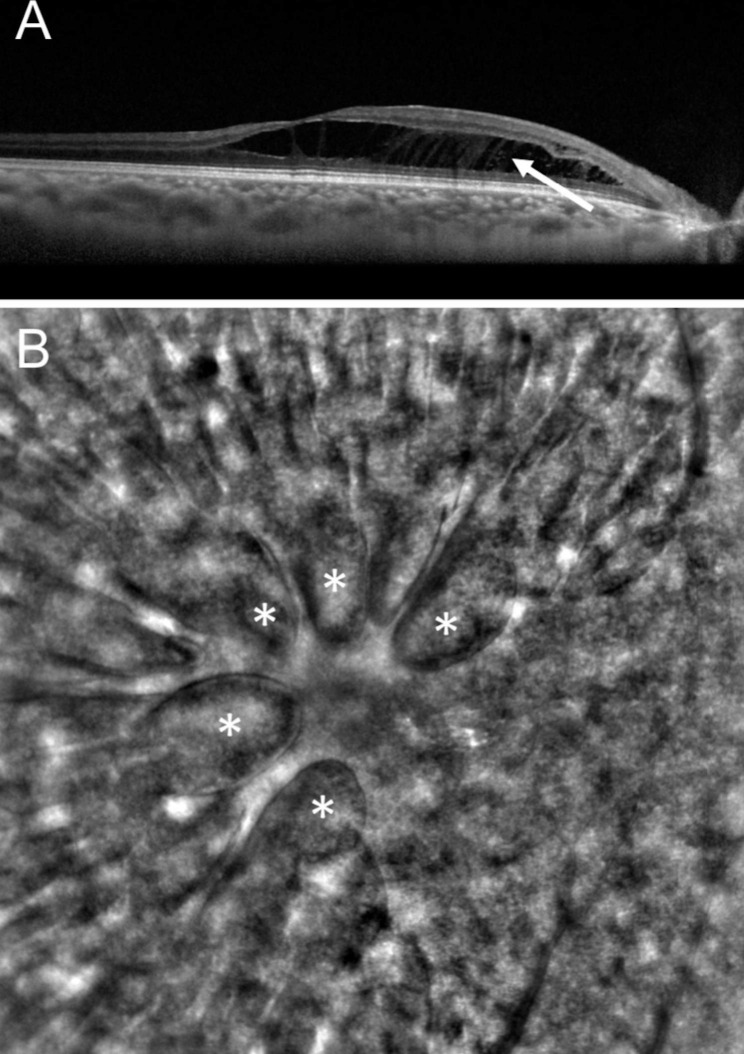
**(A)** SD-OCT scan showing macular retinoschisis (arrow), **(B)** AO scan showing a spoke-wheel pattern at the level of the retinoschisis (*)

Localized vitreomacular adhesions were present in all patients with no sign of vitreomacular traction (Fig. 1**& Supplementary Fig. 1**). AO was performed on 4 eyes (3 patients) but acquisitions were interpretable in only 2 eyes (2 patients), due to difficulties to stare at the target with the fellow glaucomatous eye and to maintain fixation during acquisition. However, it allowed en-face visualization of a LCD in one eye, which appeared as a small hyporeflective oval-shaped zone, which was confirmed with the EDI SD-OCT scan performed at the same location (Fig. 2B, C). Retinal acquisitions focusing on macular retinoschisis revealed a spoke-wheel pattern, previously described in the context of X-linked retinoschisis [12] (Fig. 3).

### Follow-up and management

Mean follow-up was 12.9 ± 9.2 months. Therapeutic management consisted in medical IOP control in 8 eyes (7 patients), while vitrectomy with internal limiting membrane (ILM) peeling and gas (C2F6) tamponade was performed in one eye, and trabeculectomy in one eye. Neither improvement or resolution of the macular retinoschisis, nor visual improvement were observed in eyes with medical treatment only or with trabeculectomy, despite significantly better IOP control at the last follow-up (14.9 ± 3.0 mmHg, *P = 0.006*). Total resolution of the macular retinoschisis was observed in the vitrectomized eye, resulting in visual improvement at last follow-up (Tables 2 and 3).

**Table 3:** Imaging characteristics and evolution of macular retinoschisis with glaucomatous optic neuropathy. OD = right eye, OS = left eye, CMT = central macular thickness, SRD = serous retinal detachment, LCD = lamina cribrosa defect, OCT = optical coherence tomography, AO = adaptive optics, NA = not applicable, ND = not done

Case #	Eye	CMT (µm)	SRD	Outer retinal hole	Visible LCD with OCT	Visible LCD with AO	Vitreo-macular adhesion	Macular retinoschisis evolution	SRD evolution
1	OS	682	+	+	-	ND	+	stable	stable
2	OD	631	+	+	-	ND	+	stable	stable
3	OD	595	-	-	-	ND	+	stable	NA
	OS	335	-	-	-	ND	+	stable	NA
4	OD	802	+	+	+	+	+	stable	increased
5	OS	721	-	-	+	ND	+	resolved	NA
6	OS	NA	+	-	+	ND	+	stable	stable
7	OD	420	-	-	-	-	+	increased	NA
	OS	377	-	-	-	-	+	stable	NA
8	OD	596	+	-	+	-	+	stable	stable

## Discussion

Our case series provides a comprehensive description of multimodal imaging characteristics of macular complications in GON. These complications were diagnosed in advanced GON in all cases. Macular retinoschisis were associated with SRD in half of the eyes, with an outer macular hole in 30% of cases. Partial vitreomacular adhesion was present in all eyes. LCD could be detected in only 40% of eyes and was always located temporally. AO was difficult to perform and did not help to detect unidentified LCD. As in the previously published literature, these complications were associated with a poor visual prognosis [8, 13, 14]. IOP lowering treatments and procedures did not result in morphological or visual improvement, while PPV was associated with resolution of the schisis and partial visual recovery.

### A diagnostic challenge

In patients with undiagnosed POAG or NTG, the presence of macular retinoschisis and/or SRD may be puzzling. As an example, case # 1 was initially misdiagnosed as a consequence of choroidal neovascularization and received three unsuccessful intravitreal anti-VEGF injections before being referred (Fig. 1A-F). In addition, LCD are barely clinically detectable in excavated discs and/or APON [15]. These findings highlight the need for multimodal imaging in these cases, where GON remains a diagnosis of exclusion after ruling out other etiologies of retinoschisis / SRD [16, 17].

### Prevalence of retinoschisis in glaucomatous eyes

Lee et al. evaluated the prevalence of retinoschisis in 372 open angle glaucomatous eyes (372 patients) and found 22 cases of peripapillary retinoschisis (6% of affected eyes) [7]. In this large series, no macular involvement was observed, probably because patients with BCVA < 20/40 were excluded. In eyes with long term follow-up (greater than one year, N = 13), the authors did not observe progression of schisis toward the macular region, suggesting that macular and peripapillary schisis may be different entities. However, in our series some patients had both macular and temporal peripapillary schisis. Altogether, with currently available data, it seems difficult to distinguish glaucoma-associated peripapillary and macular schisis as specific entities. Higher IOP and worse MD were significant risk factors for the development of peripapillary retinoschisis in glaucomatous eyes. There have been no studies reporting the prevalence of retinoschisis with macular involvement in glaucomatous eyes, probably due to their scarcity.

### Pathophysiology

The origin of retinoschisis in glaucomatous eyes has been debated in recent years. It is accepted that it may be caused by a LCD, which is frequently associated with an APON [3, 10]. However, not all LCD or APON lead to peripapillary or macular retinoschisis. Kiumehr et al. [18] and You et al. [19], respectively reported 11 and 16 glaucomatous eyes with LCD, while none presented with peripapillary / macular retinoschisis or SRD. In our series, a LCD was observed in only 40% of cases on OCT, similarly to the report of Lee et al. [7]. In a case series of 11 eyes of 11 patients who underwent pars plana vitrectomy for macular retinoschisis and GON, Inoue et al. hypothesized that macular retinoschisis could develop from vitreous traction near the retinal nerve fiber layer defect in eyes with GON and suggested that the traction on the structurally fragile RNFL contributed to the retinoschisis [13]. Vitreomacular adhesion was present in all our cases but it was not associated with observable vitreomacular traction. Besides, as incomplete posterior vitreous detachment (PVD) associated with partial vitreomacular adhesion is relatively frequent, we believe it cannot be the unique explanation for macular complications in these patients.

The origin of intra- and subretinal fluid is also a topic for discussion. Some authors have proposed a cerebrospinal origin of the fluid, passing through the LCD to reach the intra- / subretinal space [8, 10]. Nevertheless, the existence of a positive pressure gradient between the eye and the subarachnoid spaces makes this hypothesis quite unlikely [20]. Moreover, subretinal fluid proteomic analysis leans toward a vitreous origin of the subretinal fluid [21]. In patients with congenital optic disc pits, two case reports demonstrated migration of silicone oil and gas from the vitreous cavity into the subretinal space following vitrectomy [22, 23], thus confirming a communication between the vitreous cavity and subretinal space.

Lee et al. [7], reported that eyes with glaucoma and retinoschisis seem to have a higher pressure than glaucomatous eyes without retinal splitting. The authors hypothesized that highly elevated IOP could play a role in the formation of retinoschisis by enabling access of vitreous fluid into the retina. Conversely, patients reported by Inoue et al. had a normal IOP [13]. Interestingly, microcystic macular changes can occur inside the internal nuclear layer in moderate to advanced glaucoma cases, giving a retinoschisis-like appearance on OCT imaging [7, 24]. More importantly, these atrophic macular changes may facilitate the occurrence of retinal splitting. In our study, AO imaging of the macular schisis showed folds with a spoke-wheel pattern that were previously described in a patient with a juvenile X-linked foveoschisis, showing no difference in the microstructure of the retinoschisis, despite a different pathophysiology (Fig. 3) [12].

### Therapeutic management

In recent years, various therapeutic strategies have been proposed to manage retinoschisis in glaucomatous eyes. Peripapillary retinoschisis spontaneously resolves in nearly half of cases and does not cause vision loss [7, 25]. Thus, conservative management should be recommended, with close functional and structural monitoring considering the potential association between peripapillary retinoschisis and glaucoma progression [6].

For glaucoma-associated macular retinoschisis, conservative management was also chosen in 12 of the 40 reported eyes (Table 4) [1, 8, 9, 13, 14, 17, 26–28].

**Table 4:** Macular retinoschisis in glaucomatous optic neuropathy: summary of published cases. M = male, F = female, BCVA = best corrected visual acuity, IOP = intraocular pressure, SRD = serous retinal detachment, PPV = pars plana vitrectomy, ILMP = internal limiting membrane peeling, GT = gas tamponade, phaco = phacoemulsification with posterior chamber intraocular lens, laser coag = laser photocoagulation, OD = right eye, OS = left eye, SRD = serous retinal detachment, N/A = not applicable, NA = not available, One asterisk indicates that patients received glaucoma eyedrops; two asterisks indicates that patients needed a second PPV due to partially resolved retinoschisis or retinal complications

Reference	Case #	Age / Gender	Initial BCVA	Initial IOP (mmHg)	PHD before surgery	Intraoperative PHD	Associated SRD	Outer retinal hole or macula hole	Surgical treatment	Follow-up (monthts)	Macular retinoschisis evolution	SRD evolution	Final BCVA	Final IOP (mmHg)
Zumbro et al., 2007 [1]	1	14/F	20/100	50	NA	-	+	-	Trabeculectomy	6	resolved	resolved	NA	20
	2	62/M	20/200	17	-	+	+	-	PPV/ILMP/GT	6	partially resolved	partially resolved	20/40	17
	3	73/M	20/1000	15	-	+	+	-	PPV/ILMP/GT	18	partially resolved	partially resolved	20/50	15
	4	63/F	20/30	27	NA	N/A	-	-	-	NA	stable	stable	NA	NA
	5	65/M	20/25	42	NA	N/A	+	+	-	NA	NA	NA	NA	NA
Zhao et al., 2011 [17]	1	60/F	20/60	15	NA	N/A	+	+	-	5	stable	stable	NA	NA
Yoshitake et al., 2014 [9]	1	69/F	20/40	11	-	+	+	-	PPV/ILMP/GT	12	resolved	resolved	20/20	11
Maidana et al., 2014 [26]	1	36/F	20/4000	46	NA	-	+	-	cyclodiode	12	stable	stable	20/4000	15
Inoue et al., 2015 [13]	1	63/F	20/200	11*	-	+	-	-	Phaco/PPV	19	resolved	resolved	20/25	NA
	2	67/F	20/133	14*	-	+	+	+	Phaco/PPV**	26	resolved**	resolved**	20/50	NA
	3	68/M	20/200	17	-	+	+	+	Phaco/PPV/ILMP	29	resolved	resolved	20/20	NA
	4	75/M	20/50	8*	-	+	+	+	Phaco/PPV/ILMP	12	resolved	resolved	20/30	NA
	5	72/F	20/600	13	+	+	+	+	Phaco/PPV**	78	resolved**	resolved**	20/200	NA
	6	73/F	20/250	10	-	+	+	+	Phaco/PPV/ILMP	4	resolved	resolved	20/125	NA
	7	60/F	20/60	17	-	+	+	+	Phaco/PPV/ILMP	15	resolved	resolved	20/30	NA
	8	62/F	20/200	15	-	+	+	+	Phaco/PPV/ILMP	36	resolved	resolved	20/20	NA
	9	75/F	20/200	12*	-	+	+	+	Phaco/PPV/ILMP**	30	resolved**	resolved**	20/100	NA
	10	81/M	20/133	10*	-	+	+	+	PPV/ILMP**	11	resolved**	resolved**	20/33	NA
	11	71/F	20/40	14*	-	+	-	-	Phaco/PPV/ILMP	12	resolved	resolved	20/30	NA
Prinzi et al., 2015 [14]	1	69/F	20/25	NA	NA	N/A	-	-	Laser coag	14	resolved	NA	20/20	NA
	2-OD	82/F	20/30	NA	NA	N/A	-	-	Laser coag	11	resolved	NA	20/25	NA
	2-OS		20/60	NA	-	N/A	+	-	Laser coag		resolved	resolved	20/40	NA
	3-OD	83/M	20/60	NA	-	N/A	-	-	Laser coag	12	partially resolved	NA	20/50	NA
	3-OS		20/40	NA	-	N/A	-	-	Laser coag		partially resolved	NA	20/30	NA
Orazbekov et al., 2015 [27]	1	64/M	20/60	10	-	+	+	+	PPV	12	resolved	resolved	20/25	NA
Woo at al., 2018 [28]	1	58/M	20/25	27*	NA	-	-	-	Trabeculectomy	12	resolved	NA	NA	NA
Yoshikawa et al., 2018 [8]	1	NA	20/222	NA	NA	+	-	-	Phaco/PPV/ILMP/GT	60	resolved	resolved	20/22	NA
	2	65/F	20/200	NA	NA	+	+	+	Phaco/PPV/ILMP **	18	resolved**	resolved**	20/67	NA
	3	NA	20/133	NA	NA	+	+	-	PPV/ILMP/GT	16	resolved	resolved	20/33	NA
	4	NA	20/667	NA	NA	+	+	-	Phaco/PPV/ILMP/GT	9	resolved	resolved	20/200	NA
	5-OD	NA	20/100	NA	NA	+	+	+	Phaco/PPV/ILMP/GT**	123	resolved**	resolved**	20/50	NA
	5-OS	NA	20/40	NA	NA	N/A	-	-	-	80	partially resolved	NA	20/50	NA
	6	50/F	20/17	NA	NA	N/A	-	-	-	69	partially resolved	NA	20/17	NA
	7	77/F	20/200	NA	NA	N/A	+	+	-	15	partially resolved	partially resolved	20/200	NA
	8	NA	20/17	NA	NA	N/A	-	-	-	20	stable	NA	20/25	NA
	9	NA	20/25	NA	NA	N/A	-	-	-	12	stable	NA	20/29	NA
	10	NA	20/17	NA	NA	N/A	-	-	-	28	Increase	NA	20/20	NA
	11	NA	20/20	NA	NA	N/A	-	-	-	20	Increase	NA	20/20	NA
	12	NA	20/29	NA	NA	N/A	-	-	-	6	stable	NA	20/17	NA
	13	NA	20/17	NA	NA	N/A	-	-	-	8	Increase	NA	20/20	NA

In these cases, partial resolution of retinoschisis and/or SRD was reported in 3 out of 12 eyes during follow-up. The mean initial BCVA in these cases was 0.3 logMAR, and none of them showed significant visual change. In our study, conservative management was chosen in 8 out of 10 eyes, due to poor expected visual prognosis. In general, conservative management should be chosen in cases with medically controlled IOP and preserved BCVA, or on the other end of the spectrum, in end-stage glaucomatous eyes with no hope for visual recovery. In this context, oral acetazolamide could be an interesting option as in addition to decrease IOP, it enhances the RPE pump and has been used as a treatment of macular retinoschisis in X-linked retinoschisis [29].

Filtering surgery (trabeculectomy) was previously reported in 2 cases, both in the context of progressive glaucomatous neuropathy with uncontrolled IOP [1, 28]. In both cases, and conversely to our results, IOP control resulted in complete resolution of the retinoschisis after the surgery. No cases of trabeculectomy combined with another surgical procedure have been reported in literature. In one eye with uncontrolled IOP and poor visual acuity (BCVA of 2.3 logMAR), a transscleral cyclophotocoagulation procedure was performed, with a good IOP result but no improvement in BCVA or macular thickness (Table 4) [26]. Prinzi et al. performed temporal peripapillary barrier laser photocoagulations in 5 eyes with glaucoma-associated macular retinoschisis. All eyes had a pre-treatment CMT < 400 μm, and SRD was present in one eye. Complete resolution of the schisis was observed in 3 eyes, and intraretinal fluid persisted in two eyes. No VF loss was observed, and no recurrence was reported during long term follow-up (12.3 years). The mean BCVA improved after the treatment, even in the two eyes without complete resolution of the retinoschisis (Table 4) [14].

Argon laser acts primarily on the pigment epithelium and the outer retina [30]. Thus, it should not affect the most superficial layers of the retina and the RNFL. However, there are conflicting evidence on potential damages to the papillomacular bundle, some authors considering them unlikely [31], while others have reported significant visual field defect [32]. In all cases, laser should be performed cautiously, using the lowest energy as possible. Pars plana vitrectomy (PPV) with PVD was performed in 20 of 40 reported eyes (20 patients out of 37). Only one eye had a reported PVD before surgery. In most cases, PPV was combined with phacoemulsification [1, 8, 9, 13, 27]. In 16/20 PPV, internal limiting membrane peeling (ILMP) was performed during the procedure, with gas tamponade in 7 eyes (7 patients) (Table 4). Complete resolution of the retinoschisis and SRD (when present) was obtained in 18 eyes. Among these cases, a second surgical procedure was necessary in 6 eyes (6 patients). Four macular holes occurred after the first surgery (including one associated with retinal detachment, 4 patients), and resolution of the maculopathy was incomplete in 2 eyes (2 patients) [8, 13]. The ILM was not peeled in 2 of 4 secondary macular holes. A second procedure was always needed in retinoschisis with associated outer layer holes. In our series, one case was successfully treated with PPV, ILMP and gas tamponade.

**Fig. 4:**
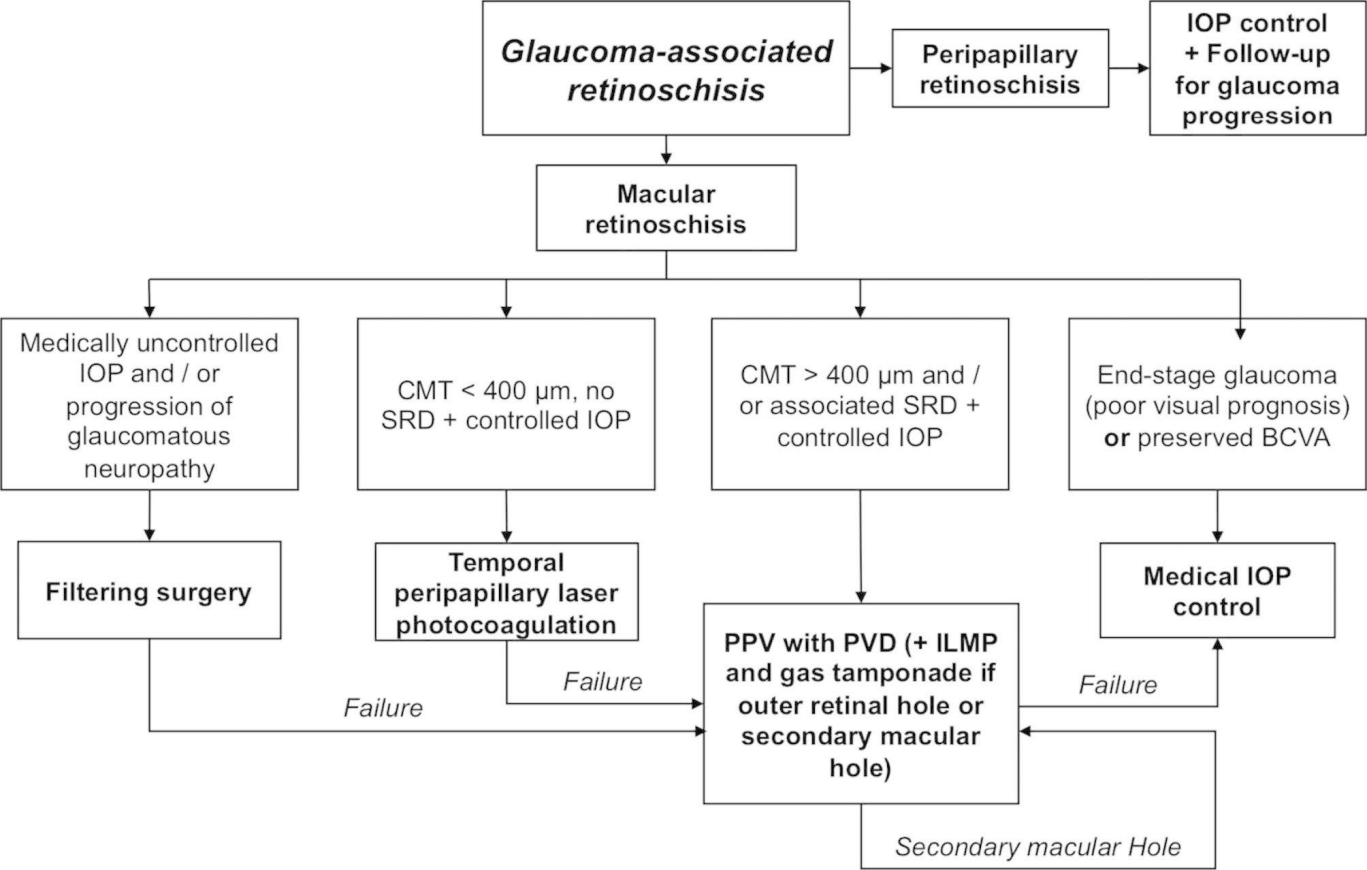
Proposed therapeutic algorithm for glaucoma-associated retinoschisis. IOP = intraocular pressure; SRD = serous retinal detachment; PPV = vitrectomy; PVD = posterior vitreous detachment; ILMP = internal limiting membrane peeling; BCVA = best corrected visual acuity

Altogether, PPV seems to be an efficient surgical treatment in most cases, especially if vitreomacular adhesions are present [13]. However, published data are not sufficient to determine if ILMP or gas tamponade are useful. In congenital optic disc related maculopathies, Avci et al. reported a successful series using gas tamponade without ILMP[33]. Rayat et al. noticed no difference in postoperative reattachment whether ILMP and/or gas tamponade was performed or not [34]. Combined juxtapapillary endolaser does not seem to influence the outcome [20, 35]. These results suggest that PPV with PVD should be performed without ILMP and gas tamponade in glaucoma with macular retinoschisis, unless an outer macular hole is observed, or in the case of re-operations. We propose a practical therapeutic algorithm based on the published literature (Fig. 4**)**.

### Limitations

The number of cases of our series is limited due to the scarcity of these retinal complications. Furthermore, despite strict inclusion/exclusion criteria, we could not completely rule out congenital optic disc pits because of the lack of available previous fundus imaging. In addition, AO did not provide useable captures in half of the tested eyes, due to a lack of fixation. Fixation is obviously impaired by a reduced VF and / or BCVA in the studied eyes and was not facilitated by the target presented to the fellow eye, which was constantly affected by advanced glaucoma (and in 2 cases by macular complications). Indeed, AO requires fixation stability during image acquisition, during at least four seconds. Finally, our therapeutic algorithm is mostly based on case series reported in the literature, and thus will require further validation.

Macular complications may occur in advanced GON and are diagnostic challenges that glaucoma and retina specialists should be aware of. High-resolution multimodal imaging may be necessary to exclude differential diagnosis and provide a better understanding of underlying involved mechanisms. Therapeutic strategy must be elaborated on a case-by-case basis, considering morphological features, IOP and visual prognosis. We propose a therapeutic algorithm which may be helpful to clinicians confronted with these situations. However, prospective evaluation of these strategies in a larger cohort of patients is warranted.

## Electronic supplementary material

Below is the link to the electronic supplementary material.

Supplementary Material 1

## Data Availability

All data are presented in the manuscript.
