# Supplementary material for: Severe macular complications in glaucoma: high-resolution multimodal imaging characteristics and review of the literature
Source: BMC Ophthalmol. 2023 Jul 14;23:318. doi: 10.1186/s12886-023-03068-z (PMC10347769; doi:10.1186/s12886-023-03068-z)
Supplement: Supplementary file 1 — Supplementary Material 1 [file 12886_2023_3068_MOESM1_ESM.docx]

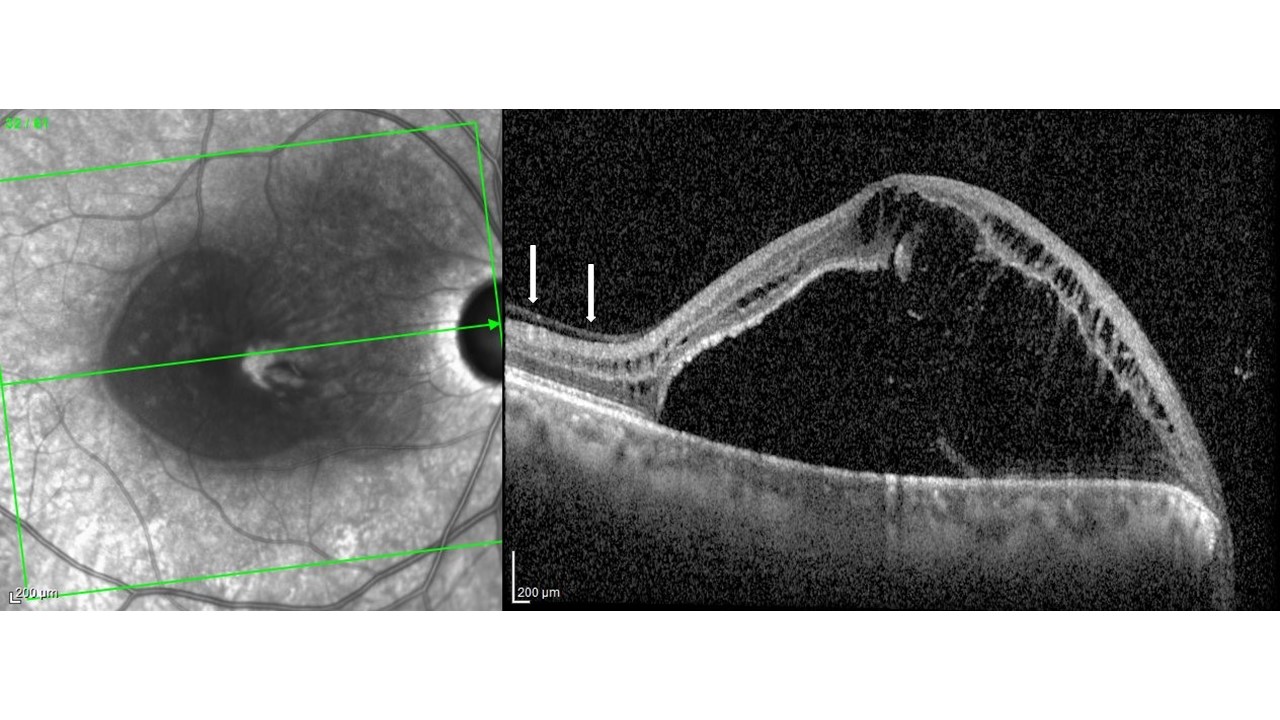


**Supplementary Figure 1 :** case #4 : the posterior hyaloid membrane is partially detached but remains parallel to the retina (arrows).
